# Mortality risk for healthcare workers and journalists in the Gaza Strip over 2023–24

**DOI:** 10.1093/eurpub/ckaf241

**Published:** 2026-01-13

**Authors:** Francesca Incardona, Federica Bellerba, Sara Gandini, Alessandro Cozzi-Lepri

**Affiliations:** EuResist Network, Rome, Italy; Department of Experimental Oncology, European Institute of Oncology, IRCCS, Milan, Italy; Department of Experimental Oncology, European Institute of Oncology, IRCCS, Milan, Italy; Centre for Clinical Research, Epidemiology, Modelling and Evaluation (CREME), Institute for Global Health, UCL, London, United Kingdom

## Abstract

Armed conflicts often expose groups protected under international humanitarian law, such as healthcare workers (HW) and journalists (JN), to disproportionate risks and in the Israel Gaza war it was particularly evident. However, quantitative assessments of their mortality relative to the general population remain limited. Using official data sources, cross-referenced lists from professional associations, and rigorous statistical methods, we estimated higher mortality among HW and JN during the 2023–24 Israel Gaza war. Mortality risks were consistently higher among these protected groups, ranging from 36% to more than sixfold higher for journalists compared with Gaza residents of the same age and sex. Our findings highlight the urgent global need to protect HW and JN in all conflict settings and to ensure accountability for violations of international humanitarian law.

## Introduction

During armed conflicts, beyond the overall civilian mortality and the mortality of frail individuals (e.g. women and children), higher mortality of healthcare workers (HW) and journalists and media workers (JN), as compared to the mortality of the general population, raise strong ethical and legal concerns. JN and HW are protected under international humanitarian law for their lifesaving role for civilians during conflicts, which exposes JN to more life-threatening conditions than the general population.

For the Israel Gaza war, formal comparisons of the risk of mortality in these protected groups versus the risk in the general population are lacking.

## Methods

In this retrospective observational study reported in accordance with STROBE guidelines, we carried out our analyses using cumulative data over two time-periods: from 7 October 2023 up to 14 October 2023 and up to 30 April 2024. We made several sensitivity analyses to account for different hypotheses and data sources.

References to Supplementary Material are provided as S1 (Data), S2 (Statistical methods), and S3_1, S3_2, S3_3 (Supplementary Tables S1–S3).

### Data sources

For the total resident population, the registered HW, the employed HW in the Gaza Strip, and for the number of Palestinian and HW deaths directly caused by the conflict, we used the data provided by the Palestinian Central Bureau of Statistics (PCBS) and the Palestinian Ministry of Health in Gaza (MoH) known to be reliable [1, 2]. We also used the estimate that approximately 30% of HW were working during the war [3].

For the total number of journalists operating in the Gaza Strip before and during the war, we obtained data from the Palestinian Journalist Syndicate, a non-Hamas organization in Gaza, member of the International Federation of Journalists (IFJ) (see Supplementary S1). For the number of deaths in this group we used the deaths lists by the Commission to Protect Journalists (CPJ) and the IFJ and created a cross-referenced list (Supplementary S3_1). We separately considered the death list by GMO [4].

### Statistical methods

To evaluate the risk of death in the protected groups relative to that of the general population, we adopted a relative risk measure introduced by Ioannidis in [5] for HW and defined as: (healthcare worker deaths/healthcare worker population)/(general population deaths/general population). We renamed it as RR_HW_ (relative risk of HW versus general population) and we extended it to JN introducing RR_JN_ (relative risk of JN versus general population).

To control for potential confounding by sex at birth we used stratification analyses. Confounding by age was minimized by restricting the calculation of the mortality in the general population to adults (defined as aged 18+).

As sensitivity analysis, we inflated the total number of deaths in the general population by an underreporting factor of 40% as suggested in [6]. Also, for the second period, we used all deaths listed in the three JN deaths lists (CPJ, IFJ and GMO), after removing duplicates. Finally, similarly to [6], we performed a capture-recapture analysis on deaths listed in the three JN lists, estimating an underreporting factor of 28% (details in Supplementary S2, S3_2, S3_3).

## Results

### Study population

The total population resident in Gaza in 2023 counts 2 226 544 individuals (1 327 019 adults, 672 347 male adults). The MoH reported 1946 cumulative deaths (1350 adults, 921 male adults) from 7 to 14 October 2023 and 34 535 cumulative deaths (23 595 estimated adults, 14 675 estimated male adults) from 7 October 2023 to 30 April 2024,  when GMO reported 19 581 adult and 9780 male adult deaths (Table 1).

**Table 1.** Risk of death due to traumatic injury in protected groups relative to the general population with 95% CI (reported data)

**Number of deaths, population size, and relative risk (RR** ^a^ **) for Healthcare Workers (HW) and for Journalists (JN) versus general population**					
	Protected population		General Population		
	Deaths	Total	Deaths	Total	**RR** ^a^ **(95% CI)**
	**7–14 October 2023**				
**Comparator—Adult Population**					
All HW (PCBS)	11	17 109	1350	1 327 019	0.64 (0.35, 1.15)
All HW (MoH)	11	7065	1350	1 327 019	1.53 (0.85, 2.77)
30% HW^b^ (PCBS)	11	5133	1350	1 327 019	2.10 (1.16, 3.81)
All JN^c^	11	1900	1350	1 327 019	5.66 (3.13, 10.23)
Active JN^c^	11	1600	1350	1 327 019	6.72 (3.72, 12.14)
**Comparator—Male Adult Population**					
All JN^c^	11	1900	921	672 347	4.21 (2.33, 7.61)
Male JN^c^	10	1600	921	672 347	4.54 (2.44, 8.45)
Active JN^c^	11	1600	921	672 347	4.99 (2.76, 9.03)
	** 7 October 2023–30 April 2024**				
**Comparator—Adult Population (MoH extrapolated** ^d^ **)**					
All HW (PCBS)	491	17 109	23 595	1 327 019	1.61 (1.47, 1.75)
All HW (MoH)	491	7065	23 595	1 327 019	3.72 (3.41, 4.06)
30% HW^b^ (PCBS)	491	5133	23 595	1 327 019	5.00 (4.59, 5.44)
All JN^c^	105	2500	23 595	1 327 019	2.31 (1.91, 2.78)
**Comparator—Male Adult Population (MoH extrapolated** ^d^ **)**					
All JN^c^	105	2500	14 675	672 347	1.89 (1.56, 2.28)
All JN (GMO)	141	2500	14 675	672 347	2.50 (2.13, 2.94)
All JN (uniquely present in the 3 JN deaths lists)	157	2500	14 675	672 347	2.77 (2.37, 3.22)
**Comparator—Adult Population (GMO April 28)**					
All HW (PCBS)	491	17 109	19 581	1 327 019	1.93 (1.77, 2.11)
All HW (MoH)	491	7065	19 581	1 327 019	4.47 (4.10, 4.87)
30% HW^b^ (PCBS)	491	5133	19 581	1 327 019	6.00 (5.51, 6.54)
All JN^c^	105	2500	19 581	1 327 019	2.77 (2.30, 3.34)
Active JN^c^	105	2200	19 581	1 327 019	3.13 (2.60, 3.78)
All JN (GMO)	141	2500	19 581	1 327 019	3.67 (3.12, 4.31)
**Comparator—Adult Male Population (GMO April 28)**					
All JN^c^	105	2500	9780	672 347	2.81 (2.33, 3.39)
Male JN^c^	94	2200	9780	672 347	2.86 (2.34, 3.49)
Active JN^c^	105	2200	9780	672 347	3.18 (2.63, 3.83)
All JN (GMO)	141	2500	9780	672 347	3.72 (3.17, 4.38)

a Ioannidis metrics: RR_HW_ or RR_JN_.

b Ministry Source cited in [4].

c Cross-Referenced list (Supplementary S3_1).

d Extrapolated assuming the same proportion observed in the subset of identified cases provided by the MoH (see Supplementary S1).

GMO, Government Media Office; HW, Health Care Workers; JN, Journalists; MoH, Ministry of Health; PCBS, Palestinian Central Bureau of Statistics.

The HW registered in Gaza in 2020 were 17 109, those employed 7065 and the estimated HW at work during the war 5133 (30% of the registered ones). The cumulative reported deaths among HW were 11 at the first and 491 at the second time point.

At the beginning of the war, there were approximately 1900 JN in the Gaza Strip, including 300 retired senior journalists; this number increased to approximately 2500 active in the Strip until April 2024, including temporary and part-time JN. The cumulative reported deaths among JN were 11 at the first time-point and 105 at the second one (141 according to GMO).

Overall, for HW at the beginning of the war, we found no clear evidence for a difference in mortality risk compared to the general adult population. In contrast, after almost seven months of war, despite our conservative assumptions, the risk of death was much higher than that of the adult population. Specifically, as of 14 October 2023 the RR_HW_ ranged between 0.64 (95% Confidence Interval [CI]: 0.35, 1.15) and 2.10 (CI: 1.16, 3.81), depending on the source and comparator group used and the exact-assumptions made (Table 1). As of 30 April 2024, the RR_HW_ ranged between 1.61 (CI: 1.47, 1.75) and 5.00 (CI: 4.59, 5.44).

The results in terms of RR_JN_ were even more concerning with estimates at the first time point which ranged between 4.21 (CI: 2.33, 7.61) and 6.72 (CI: 3.72, 12.14), and at the second time point, between 2.31 (CI: 1.91, 2.78) and 3.72 CI: (3.17, 4.38), Table 1. Table 2 shows results after accounting for underestimation of mortality in the general population. When accounting also for under-reporting of deaths in JN, our main analysis for the second time point shows a striking RR_JN_ of 2.51 (CI: 2.20–2.87). Unlike what was observed for the HW, the higher relative mortality risk in the JN was evident from the beginning of the conflict and showed a stable and only slightly declining trend up to 30 April 2024. Importantly, Supplementary Table S3_1, shows that out of 105 journalists’ deaths, 58 (55%) happened at home.

**Table 2.** Risk of death due to traumatic injury in protected groups relative to the general population with 95% CI (after correcting the number of deaths in general population for under-reporting)

**Number of deaths, population size, and relative risk (RR** ^a^ **) for Healthcare Workers (HW) and for Journalists (JN) versus general population**					
	Protected population		General Population		
	Deaths	Total	**Deaths** ^b^	Total	**RR** ^a^ **(95% CI)**
	**7–14 October 2023**				
**Comparator—Adult Population**					
All HW (PCBS)	11	17 109	1890	1 327 019	0.45 (0.25, 0.82)
All HW (MoH)	11	7065	1890	1 327 019	1.09 (0.60, 1.98)
30% HW^c^ (PCBS)	11	5133	1890	1 327 019	1.50 (0.83, 2.72)
All JN^d^	11	1900	1890	1 327 019	4.05 (2.24, 7.31)
Active JN^d^	11	1600	1890	1 327 019	4.80 (2.66, 8.67)
**Comparator—Male Adult Population**					
All JN^d^	11	1900	1289	672 347	3.01 (1.66, 5.43)
Male JN^d^	10	1600	1289	672 347	3.24 (1.75, 6.03)
Active JN^d^	11	1600	1289	672 347	3.57 (1.97, 6.44)
	**7 October 2023–30 April 2024**				
**Comparator—Adult Population (MoH extrapolated** ^e^ **)**					
All HW (PCBS)	491	17 109	33 033	1 327 019	1.15 (1.06, 1.26)
All HW (MoH)	491	7065	33 033	1 327 019	2.68 (2.45, 2.92)
30% HW still at work^c^ (PCBS)	491	5133	33 033	1 327 019	3.59 (3.30, 3.91)
All JN^d^	105	2500	33 033	1 327 019	1.66 (1.38, 2.00)
**Comparator—Male Adult Population (MoH extrapolated)**					
All JN^d^	105	2500	20 545	672 347	1.36 (1.13, 1.64)
All JN (GMO)	141	2500	20 545	672 347	1.80 (1.53, 2.12)
All JN (uniquely present in the 3 JN deaths lists)	157	2500	20 545	672 347	1.99 (1.71, 2.32)
All JN (including 28% unlisted for under-reporting)^f^	201	2500	20 545	672 347	2.51 (2.20, 2.87)
**Comparator—Adult Population (GMO April 28)**					
All HW (PCBS)	491	17 109	27 413	1 327 019	1.39 (1.27, 1.51)
All HW (MoH)	491	7065	27 413	1 327 019	3.21 (2.95, 3.50)
30% HW^c^ (PCBS)	491	5133	27 413	1 327 019	4.31 (3.96, 4.70)
All JN^d^	105	2500	27 413	1 327 019	1.99 (1.65, 2.40)
Active JN^d^	105	2200	27 413	1 327 019	2.25 (1.87, 2.71)
All JN (GMO)	141	2500	27 413	1 327 019	2.64 (2.25, 3.10)
**Comparator—Adult Male Population (GMO April 28)**					
All JN^d^	105	2500	13 692	672 347	2.02 (1.67, 2.44)
Male JN^d^	94	2200	13 692	672 347	2.05 (1.68, 2.50)
Active JN^d^	105	2200	13 692	672 347	2.28 (1.89, 2.75)
All JN (GMO)	141	2500	13 692	672 347	2.68 (2.28, 3.14)

a Ioannidis metrics: RR_HW_ or RR_JN_.

b Including 40% unlisted for under-reporting as per [1] (see Supplementary S2).

c Ministry Source cited in [4].

d Cross-Referenced list (Supplementary S3).

e Extrapolated assuming the same proportion observed in the subset of identified cases provided by the MoH (see Supplementary S1).

f Evaluated by capture-recapture analysis on the 3-list source (see Supplementary S2).

GMO, Government Media Office; HW, Health Care Workers; JN, Journalists; MoH, Ministry of Health; PCBS, Palestinian Central Bureau of Statistics.

## Discussion

We found a consistently higher relative mortality risk among both examined protected groups, particularly among JN, as compared to the general population. Our analyses were robust to sensitivity assumptions. In Table 2, the under-reporting corrective factor was applied to the general population deaths but not to the HW deaths and therefore these are likely to be conservative estimates of the relative risks. Noteworthy, in Table 1, the relative risk for HW in the second period appeared to be higher than that observed in other conflicts (1.38 in Syria 2011–24) [5].

Our analysis has limitations that need to be acknowledged. The biggest limitation is the intrinsic difficulty in acquiring mortality reports in ongoing conflicts. However, the data sources used represent the most comprehensive and transparent records currently accessible. We partly used estimated data, including predictions from statistical methods as done by others [6]. The assumption that 30% of the total HW were at work during the war [3] is consistent with the progressive disruption of the health system during the conflict [7].

Our epidemiological metric, as originally proposed [5], is a crude measure of relative risk mortality. However, we attempted to minimize potential confounding by age and sex by stratification and by restricting some of the analyses to only-adult-male populations or to only-active (not retired) JN.

Although the war in Gaza shows numbers of HW and JN casualties that far exceed those in other wars [8], the CPJ and the United Nations denounce increased attacks on HW and JN around the world (see Supplementary S1). The Israel Gaza conflict exemplifies how the erosion of protections for these groups can amplify civilian suffering. Indeed, deaths among HW are likely to lead to reduced healthcare support and increased mortality, including from cancer [9], while deaths among JN disrupt the “early warning mechanism” for genocide and other core international crimes [10], which is critical for raising global awareness and informing policy decisions.

## Supplementary Material

ckaf241_Supplementary_Data

## Data Availability

All data supporting the findings of this study are available within the manuscript and its supplementary materials.
