# Supplementary material for: Mortality risk for healthcare workers and journalists in the Gaza Strip over 2023–24
Source: Eur J Public Health. 2026 Jan 13;36(2):ckaf241. doi: 10.1093/eurpub/ckaf241 (PMC13064847; doi:10.1093/eurpub/ckaf241)
Supplement: ckaf241_Supplementary_Data [file ckaf241_supplementary_data.docx]

**Supplementary material for the short communication “Mortality risk for health-care workers and journalists in the Gaza Strip over 2023-2024”**

European Journal of Public Health

Authors: Francesca Incardona, Federica Bellerba, Sara Gandini, Alessandro Cozzi-Lepri

Corresponding Author:

Sara Gandini, PhD

European Institute of Oncology

Email: [sara.gandini@ieo.it](mailto:sara.gandini@ieo.it)

Table of content:

[**S1. Data 1**](#_heading=h.vuar4z3mybqd)

[Total population in the Gaza Strip 1](#_heading=h.6p0p7lo3zvy0)

[Palestinians deaths directly caused by the conflict in the Gaza Strip 2](#_heading=h.qhd51o2tkj8k)

[Total journalists and media workers in the Gaza Strip 3](#_heading=h.qjog6a9qxh0)

[Journalists deaths directly caused by the conflict in the Gaza Strip 4](#_heading=h.ttxuoce1eboi)

[Total HWs in the Gaza Strip 5](#_heading=h.4k1sov7zjl6r)

[HWs deaths directly caused by the conflict in the Gaza Strip 5](#_heading=h.bd1pxonix48c)

[**S2. Statistical methods 5**](#_heading=h.ac18omh6hby)

[**References for the supplementary material 7**](#_heading=h.5dzc8xr1x2ej)

[**S3_1. Table S1. Cross-referenced list of Palestinian journalists killed in Gaza,
according to at least two reliable sources 8**](#_heading=h.z5e5wucz754)

[**S3_2. Table S2. Cross-Tabulation between journalist lists 14**](#_heading=h.v0pcwcebn9m6)

[**S3_3. Table S3. Estimates from fitting a log-linear model on the counts 15**](#_heading=h.njc76j18h8l5)

# S1. Data

Locating sources and evaluating them against available evidence and criticism has been a fundamental part of this study. In particular, the total number of journalists is not publicly available data and required in field research to identify and reach a trustable source.

## Total population in the Gaza Strip

For the total population we considered the figures provided by the Palestinian Central Bureau of Statistics (PCBS),^1^ a government office. The CIA World Factbook^2^ yields similar numbers, 3·8% lower.
In a prudent approach and also for consistency, we adopted the PCBS figure: a total population of 2,226,544 individuals in 2023.
Children make up 40·4% of the population.

## Palestinians deaths directly caused by the conflict in the Gaza Strip

For the number of Palestinian deaths by traumatic injuries directly caused by the conflict, we considered the data provided by the Palestinian Ministry of Health in Gaza (MoH). Reliability of the source has been ascertained early in the conflict and in previous conflicts.^3,4^ In the first months of the war, data were compiled from reports of deaths at hospitals, morgues and ambulance services. The data from the MoH coincide with data from the UN Office for the Coordination of Humanitarian Affairs (OCHA), with minor differences which disappear when a seven-day moving average is applied.

For the analysis on October 14, 2023, we used the report issued by the MoH on the period October 7-26, 2023^5^ with the names and identity card numbers of the dead. The report indicated day by day the total deaths and the breakdown by males/females and adults/children. We calculated the percentage of adults over the males each day (or the percentage of males over the adults) and calculated a total of 921 male adult deaths on 14 October, out of 1350 adults and a total of 1,946 fatalities.

In November 2023 the MoH declared that it could not properly report deaths because of the disruption to most of the hospitals in the northern Gaza Strip. In December, the MoH started counting data using a combined method including “reliable media sources”, in collaboration with the Government Media Office (GMO). Critics of this method suggest that by using the combined method, the MoH data could underestimate male casualties or overestimate the number of females and children. The OCHA decided from May 8, 2024 to report the breakdown of males, females and children only on the subset of deaths recorded by the MoH via the hospitals-morgues-ambulances route and fully identified: “Previous data breakdowns provided in this snapshot of women and children killed were attributed by the GMO based on reported fatalities. The fatality breakdowns currently cited are those that the MoH in Gaza has recently fully identified out of the higher number of casualties they report.” (https://www.ochaopt.org/content/hostilities-gaza-strip-and-israel-reported-impact-day-220)

For the analysis of the data up to April 30, 2024, we used the data from the MoH Emergency report^6^ issued on April 30, 2024, which reports 34,535 total deaths, including 24,127 fully identified people. The report indicated that 31·7% of the 24,127 subset of fully identified deaths were children, and 62·2% were males. We applied the same ratios to total deaths. The percentage of adult male deaths over the total number of identified deaths was calculated by subtracting the percentage of child deaths from the total number of male deaths. Thus, we estimated 68·3% of the total deaths to be adults (= 23,595), and 42·5% of the total deaths to be male adults (68·32% of the total male deaths = 14,675). It may be noted that in the September Report^7^ issued by the MoH on September 15, 2024, fully identifying the 34,344 dead up to August 31, 2024, 26·88% are of children under 15 and 33·06% are under 18, in line with the April Report^6^.

For further sensitivity analysis, we used data from the GMO report^8^ of April 28, 2024 which gives 34,454 deaths, 14,873 children and 9,801 women; i.e. a total of 9,780 male adult deaths.

## Total journalists and media workers in the Gaza Strip

Since the information about the total number of journalists working in the Gaza Strip during our period of observation was not publicly available, we approached the Palestinian Ambassador in Italy, the International Federation of Journalists (IFJ), several Palestinian and Italian journalists and associations and finally the Palestinian Journalists’ Syndicate (PJS). We obtained figures from Dr. Tahseen Al Astal, the PJS Deputy Secretary, by means of a series of written interviews he gave us from Gaza between 29 March and 21 April 2024. The PJS is a non-Hamas organisation in Gaza and an official member of the International Federation of Journalists (IFJ). Al Astal writes: “The number of journalists registered with the union is 1,600. Membership is divided between working members, temporary members, and honorary members, senior retired journalists.” Of these 1,600, 300 were women, 1,323 were active journalists who were invited to the General Conference and Replacement of Fellows held in May 2023 (of these, some 1,000 journalists worked for newspapers and the others were freelance). The remaining members were retired or honorary fellows. There were around 300 Palestinian journalists in the Gaza Strip who were not members of the Syndicate before the conflict, giving a total of 1,900 journalists and media workers present in the Gaza Strip at the beginning of the war, including senior retired journalists.
During the war, as Al Astal writes, “The number of journalists and media workers has naturally increased significantly, with many media companies and institutions seeking the help of recent media college graduates”, partly because “the Israeli occupation does not allow foreign journalists to enter the Gaza Strip.”
“According to the data, information and statistics available to us”, clarified Al Astal on April 21, 2024 “approximately 2,500 journalists and media workers have worked during the war. This number includes 1,321 registered with the union and active workers, as well as those who are not affiliated with the union for political reasons, those who hold an emergency identification card because they do not meet the conditions for membership, and those who worked for short periods due to war conditions and the absence of foreign press”. This last group of temporary and part-time media workers may reach “350 people, but is not exactly specified”. “The Journalists’ Syndicate”, adds Al Astal, “counts all journalists and includes all journalists in its services, and defends every journalist, members of the syndicate and non-members alike, given that membership is voluntary.”

Dr Al Astal confirmed this report on May 25, 2024.

There is no other journalists’ syndicate in the Gaza Strip.

The overall picture was backed up by information gathered from independent journalists working in the area, whom we interviewed: an Italian journalist based in Jerusalem; Safwat Kahlout, Palestinian journalist from the Gaza Strip, staff of Al Jazeera English; Haggai Matar, executive director of the Israeli +972 Magazine; Jonathan Dagher, Head of the Middle East desk at Reporters Without Borders; and Tim Dawson, Deputy General Secretary of the International Federation of Journalists.

In accordance with the report from Al Astal, we used 2,500 as the total number of journalists working in Gaza after almost seven months of conflict, which includes people who worked for short periods of time. With a prudent approach, we considered 2,500 as the maximum denominator.

## Journalists deaths directly caused by the conflict in the Gaza Strip

The Committee to Protect Journalists (CPJ) has a curated database, accessible and downloadable, of the number of journalists and media workers killed worldwide since 1992.^9^ CPJ researchers independently investigate and verify the circumstances behind every death. For each journalist there is a short resumé of their affiliations, the circumstances of their death and the sources of the information.
With reference to the journalists killed in Israel and the Occupied Palestinian Territory, the CPJ warns that there might be delays in its publication of cases.
On January 20, 2025, the CPJ lists 106 Palestinian journalists and media workers killed and verified over the period October 7, 2023 - April 30, 2024.

The IFJ keeps a list of journalists killed in Gaza,^10^ compiled “working closely with its affiliate, the Palestinian Journalists' Syndicate (PJS) to verify information in real time”. For each journalist, it also gives a short resumé of affiliations, circumstances of death and sources. On January 20, 2025, the IFJ lists 104 Palestinian journalists and media workers killed in the period October 7, 2023 - April 30, 2024.

We compared the two lists, which are clearly based on independent information, with names transliterated differently from Arabic into Latin script, we checked other sources, like Reporters Without Borders,^11^ and compiled our own cross-referenced list , including only the cases for which at least two independent reliable sources were available. The resulting cross-referenced list is available below as Table S1, and amounts to 105 cases in the period October 7, 2023 - April 30, 2024.

For the sensitivity analyses, we also considered the list provided by the GMO^8^ On April 28, 2024, reporting a total of 141 journalists killed up to that date.

Finally, we used the CPJ, IFJ and GMO lists for a 3-list capture-recapture analysis (see S2. Statistical methods), where the total number of unique deaths present in the 3-list source was 157. We estimated that the number of deceased journalists could be under-reported by a factor of 28% and the total number of journalists’ deaths including the unlisted could be up to 201 deaths.

The war in Gaza shows a number of JN casualties per year that far exceeds that of any other war studied since the 20th century.^12^ However, the CPJ reports abuses and crimes against journalists in many countries around the world.

## Total HWs in the Gaza Strip

For the population of HWs we used two sources: the PCBS database, 2001-2020, which reports 17,109 registered doctors and nurses in the Gaza Strip in 2020; and the 2021 Annual report of the MoH of Gaza, which lists 7,065 doctors and nurses employed in the Gaza Strip in 2020, implying that only a fraction of the registered HWs are actually employed as such.

We also used the 30% estimate made by Ashraf al-Qudra, spokesperson for the Gaza Health Ministry, as reported by Berger et al: “Only about 30 percent of the Gaza medical staffers who worked at hospitals and clinics before the war are still on duty, either because they’ve had to flee their homes or because they fear moving around the Strip”.^13^
This estimate is consistent with the progressive disruption of healthcare observed in the Gaza strip during the war: a geospatial anlysis carried out on November 7, 2023, shows that “approximately 9% of non-medical buildings and medical complexes alike sustained damage during the first month of the bombardment”^14^; OCHA reported on May 1, 2024^15^ that 24 hospitals were out of service, 12 hospitals were partially functioning, and 80% of primary healthcare centres were not operating.

## HWs deaths directly caused by the conflict in the Gaza Strip

For the deaths among HW until October 14, 2023, we consider the data from the OCHA report on 14 October^16^ of 11 HW deaths.

For the second time point, we used the data from the MoH Emergency report^6^ issued on April 30, 2024, which reports the death of “more than 491 staff and medical professionals”.

; It has to be noted that the assault on Gaza’s health system tops the list with which the United Nations denounces that “health and aid workers are targeted in conflicts around the world”^17^.

# S2. Statistical methods

Mortality risks were calculated as the number of deaths by traumatic injury divided by the number of residents in the general population or in the identified groups.

To evaluate the risk of death in the HW relative to that of the general population, we adopted the a metrics proposed by Ioannidis^18^ for HW: the healthcare worker versus population hazard, (HPH) expressed as a a relative risk measure (HPH(r)) and defined as: (healthcare worker deaths/healthcare worker population)/(general population deaths/general population). To increase clarity we renamed it as RR_HW_ (relative risk of HW vs. general population) and extended it to JN introducing the RR_JN_ (the relative risk of JN vs. general population), defined similarly. A RR equal to 1 indicates a risk of death for the vulnerable population the same as that of the general population; while RR greater (lower) than 1 indicates a risk for the vulnerable population higher (smaller) than that of the general population.

In our analysis, we aimed to control for potential confounding by age and sex at birth using stratification.

Several sensitivity analyses were performed, incorporating different data sources and testing different hypotheses.

We extrapolated the number of deaths among the adult and the adult male population in Gaza by assuming the same proportional distribution of deaths between adults/children and males/females observed in the identified cases provided by the MoH. The percentage of adult male deaths over the total number of identified deaths was calculated by subtracting the percentage of child deaths from the total number of male deaths.

In sensitivity analyses (Table 1B), we inflated the total number of deaths in the general population by a factor of 40%. This correction factor is based on the work by Jamaluddine et al^19^ where the authors estimated the true number of deaths in the general population in Gaza, after accounting for under-reporting due to governmental office disruption.

In the analysis regarding journalists, given the predominance of male journalists, besides the analysis using the whole general population as the comparator, we also restricted the denominator to the male adult population alone. In addition, we performed analyses specifically comparing male journalists to male adults. We carried out comparisons excluding retired journalists to focus solely on active professionals. Also, we used all deaths listed in the three lists (the CPJ, the IFJ and the GMO lists), after removing duplicates.

Finally, we adopted a similar approach as Jamaluddine et al^19^ to correct the number of cumulative deaths among JNs by April 30, 2024. In detail, we performed a capture-recapture analysis on a dataset which included the deaths listed in the three lists ( CPJ, IFJ and GMO). To build this dataset (the 3-list source), first, we checked and standardised the spelling of Arabic names in the three lists and applied a multistep approach to remove duplicates. If the date of death was missing, we used multiple imputation to fill the missing data. Then, we identified deaths which were solely listed by one source and deaths which were listed in more than one source. We therefore evaluated the number of unique deaths present in the 3-list source. Finally, we fitted a log-linear model containing terms for the probability of appearing on the CPJ list, on the IFJ list and on the GMO list. Because the information regarding the deaths were acquired by the organisations from similar original sources, and therefore could not be considered as independent, we also included in the model the two-way interactions terms among the CPJ list and the GMO list and among the CPJ list and the IFJ list. In an alternative multinomial logit regression model performed on individual data we controlled for the exact date of death and sex at birth to help account for individual heterogeneity and predictions were similar. The log-linear model is tabulated in Table S2 (S3_2), fitted on the cross-tabulated frequency counts reported in Table S3 (S3_3). It provides a total of 201 deaths, with 44 additional deaths not listed in any of the three sources.

For the analysis on healthcare workers, we compared their risk of death to that of the entire adult population, assuming a more balanced distribution between males and females within the profession.

All RR_HW_ and RR_JN_ estimates and corresponding 95%CI were tabulated after stratification by time periods (first week of war and until end of April 2024) with and without the correction for under-reporting.

All analyses were carried out using SAS software V9.4 (SAS Institute Inc., Cary, NC, USA).

# References for the supplementary material

1. Palestinian Central Bureau of Statistics <https://www.pcbs.gov.ps>. Accessed May 26, 2024
2. The CIA World Factbook [https://www.cia.gov/the-world-factbook/countries/gaza-strip/#people-and-society](https://www.cia.gov/the-world-factbook/countries/gaza-strip/%23people-and-society) Accessed May 26, 2024
3. Huynh BQ, Chin ET, Spiegel PB. No evidence of inflated mortality reporting from the Gaza Ministry of Health*. The Lancet.* 2023;403(10421):23-24. doi:10.1016/S0140-6736(23)02713-7
4. Jamaluddine Z, Checchi F, Campbell Oona M R. Excess mortality in Gaza: Oct 7–26, 2023*. The Lancet*. 2023;402(10418):2189-2190. doi:10.1016/S0140-6736(23)02640-5
5. Palestinian Ministry of Health. Detailed report - For the victims of the Israeli aggression on the Gaza Strip During the period (7-26 October 2023) (<https://site.moh.ps/>) (Over the course of the project the MoH website became unavailable. The report can be found at: <https://www.palestine-studies.org/en/node/1654513> ). Accessed May 26, 2024.
6. Palestinian Ministry of Health. Health sector emergency report - For the 206th day of aggression - Monday, 29 April 2024. [https://web.telegram.org/k/#@MOHMediaGaza](https://web.telegram.org/k/%23@MOHMediaGaza) Accessed May 26, 2024.
7. Palestinian Ministry of Health in Gaza. Martyrs until 31-08-2024. [https://web.telegram.org/k/#@MOHMediaGaza](https://web.telegram.org/k/%23@MOHMediaGaza) Accessed September 16, 2024.
8. GMO Report 28 April 2024 on Telegram <https://t.me/mediagovps/2770> , Accessed January 20, 2025.
9. CPJ. Explore CPJ's database of attacks on the press. <https://cpj.org/data/> Accessed January 20, 2025.
10. IFJ. War in Gaza: Journalists are under attack. <https://www.ifj.org/war-in-gaza>. Accessed January 20, 2025.
11. Reporters Without Borders. Israel-Gaza war: list of journalists killed in the line of duty in Palestine, Israel and Lebanon gets longer <https://rsf.org/en/israel-gaza-war-list-journalists-killed-line-duty-palestine-israel-and-lebanon-gets-longer> Accessed January 20, 2025.
12. Turse N. News Graveyards: How Dangers to War Reporters Endanger the World. Brown University April 1, 2025. chrome-extension://efaidnbmnnnibpcajpcglclefindmkaj/<https://costsofwar.watson.brown.edu/sites/default/files/papers/Turse_Costs-of-War_The-Reporting-Graveyard-4-2-25.pdf>
13. Berger M, Tarnopolsky N, Fisher M, Booth W. Calls rising globally for pause in Israel-Gaza violence to let aid arrive. *Washington Post.* October 25, 2023. <https://www.washingtonpost.com/world/2023/10/25/israel-gaza-hamas-war-turkey/> Accessed May 26, 2024.
14. Poole DN, Andersen D, Raymond NA, et al. Damage to medical complexes in the Gaza Strip during the Israel–Hamas war: a geospatial analysis. BMJ Glob Health 2024;9:e014768. doi:10.1136/bmjgh-2023-014768
15. OCHA report day 208, May 1, 2024. <https://www.ochaopt.org/content/hostilities-gaza-strip-and-israel-reported-impact-day-208> Accessed January 20, 2025.
16. OCHA report 14 October 2023 <https://www.ochaopt.org/content/hostilities-gaza-strip-and-israel-flash-update-8> Accessed May 26, 2024.
17. UN News. Health and aid workers targeted in conflicts around the world, UN agency says, August 23, 2025 https://news.un.org/en/story/2025/08/1165710 . Accessed October 17, 2025.
18. Ioannidis JPA, Differential risk of healthcare workers versus the general population during outbreak, war and pandemic crises. Eur J Epidemiol. 2024; doi:10.1007/s10654-024-01169-7
19. Jamaluddine Z, Abukmail H, Aly S, Campbell O M R, and Checchi F. Traumatic injury mortality in the Gaza Strip from Oct 7, 2023, to June 30, 2024: a capture–recapture analysis. The Lancet January 09, 2025 DOI:[10.1016/S0140-6736(24)02678-3](https://doi.org/10.1016/S0140-6736(24)02678-3)

# S3_1. Table S1. Cross-referenced list of Palestinian journalists killed in Gaza, according to at least two reliable sources

| **Date of death** | **Status (CPJ)** | **Full name** | **Sources** | **Sex** | **How/ where** | **Type of Employment** | **Working for** |
| --- | --- | --- | --- | --- | --- | --- | --- |
| 7/10/2023 | C | Ibrahim Mohammad Lafi | CPJ - IFJ | M | Shot | Staff | Ain Media |
| 7/10/2023 | C | Mohammad Jarghoun | CPJ - IFJ - UNESCO - BBC | M | Shot § | Staff | Smart Media |
| 7/10/2023 | C | Mohammed Al-Salhi | CPJ - IFJ | M | Home | Staff | Fourth Authority news agency |
| 9/10/2023 | C | Hisham Alnwajha | CPJ - IFJ | M | Airstrike | Staff | â€œKhabarâ€ News Agency |
| 9/10/2023 | C | Mohammed Sobh | CPJ - IFJ | M | Airstrike | Staff | â€œKhabarâ€ News Agency |
| 9/10/2023 | C | Saeed al-Taweel | CPJ - IFJ | M | Airstrike | Staff | Al-Khamsa News |
| 11/10/2023 | C | Mohamed Fayez Abu Matar | CPJ - IFJ | M | Airstrike | Freelance |  |
| 12/10/2023 | C | Ahmed Shehab | CPJ - IFJ | M | Home | Staff | Sowt Al-Asra Radio (Radio Voice of the Prisoners) |
| 13/10/2023 | C | Husam Mubarak | CPJ - IFJ | M | Airstrike | Staff | Al Aqsa Radio |
| 13/10/2023 | C | Salam Mema | CPJ - IFJ | F | Home | Freelance | Palestinian Media Assembly - Al Quds radio |
| 14/10/2023 | C | Yousef Maher Dawas | CPJ - IFJ | M | Home | Freelance | Palestine Chronicle and We Are Not Numbers (WANN) |
| 16/10/2023 | C | Abdulhadi Habib | CPJ - IFJ | M | Home | Staff | Al-Manara News Agency, HQ News Agency |
| 17/10/2023 | C | Issam Bhar | CPJ - IFJ | M | Home | Staff | Al-Aqsa TV |
| 17/10/2023 | C | Mohammad Balousha | CPJ - IFJ | M | Airstrike | Staff | Palestine Today |
| 18/10/2023 | C | Sameeh Al-Nady | CPJ - IFJ | M | Home | Staff | Al-Aqsa TV |
| 19/10/2023 | C | Khalil Abu Aathra | CPJ - IFJ | M | Airstrike | Staff | Al-Aqsa TV |
| 20/10/2023 | C | Mohammed (Abu) Ali | CPJ - IFJ | M | Home | Staff | Al-Shabab Radio (Youth Radio) |
| 22/10/2023 | C* | Roshdi Sarraj | CPJ - IFJ | M | Airstrike | Freelance | Ain Media |
| 23/10/2023 | C | Mohammed Imad Labad | CPJ - IFJ | M | Airstrike | Staff | Al Resalah |
| 24/10/2023 | Killed | Ibrahim Marzouq | CPJ - Institute for Palestine Studies - Palestine Today | M | Home | Staff | Palestine Today TV |
| 25/10/2023 | C | Ahmed Abu Mhadi | CPJ - IFJ | M | Home | Staff | Al-Aqsa TV |
| 25/10/2023 | C | Jamal Al-Faqaawi | CPJ - IFJ | M | Home | Staff | Mithaq Media Foundation |
| 25/10/2023 | C | Saed Al-Halabi | CPJ - IFJ | M | Home | Staff | Al-Aqsa TV |
| 25/10/2023 | C | Tasneem Bkheet | CPJ - SKeies | F | Home | Freelance | Al Saada |
| 26/10/2023 | C | Duaa Sharaf | CPJ - IFJ | M | Home | Staff | Radio Al-Aqsa |
| 27/10/2023 | C | Yasser Abu Namous | CPJ - IFJ | M | Home | Staff | Al-Sahel |
| 30/10/2023 | Killed | Nazmi Al-Nadim | CPJ - IFJ | M | Home | Staff | Palestine TV |
| 31/10/2023 | Killed | Imad Al-Wahidi | CPJ - IFJ | M | Home | Staff | Palestine TV |
| 31/10/2023 | Killed | Majed Kashko | CPJ - IFJ | M | Home | Staff | Palestine TV |
| 1/11/2023 | C | Iyad Matar | CPJ - IFJ | M | Airstrike | Staff | Al-Aqsa TV |
| 1/11/2023 | C | Majd Fadl Arandas | CPJ - IFJ | M | Airstrike | Staff | Al-Jamaheer |
| 2/11/2023 | C | Mohamad Al-Bayyari | CPJ - IFJ | M | Airstrike | Staff | Al-Aqsa TV |
| 2/11/2023 | C | Mohammed Abu Hatab | CPJ - IFJ | M | Home | Staff | Palestine TV |
| 7/11/2023 | C | Mohamed Abu Hassira | CPJ - IFJ | M | Home | Staff | WAFA |
| 7/11/2023 | C | Yahya Abu Manih | CPJ - IFJ | M | Airstrike | Staff | Al-Aqsa Radio |
| 10/11/2023 | C | Ahmed Al-Qara | CPJ - IFJ - PJS - Al-Dostor | M | Targeted | Freelance |  |
| 12/11/2023 | Killed | Moussa Al-Borsh | CPJ - IFJ - SKeyes - MADA | M | Home | Staff | Namaa Radio |
| 13/11/2023 | C* | Ahmed Fatima | IFJ - PJS - Ahram - RSF | M | Airstrike | Staff | Al Qahera News TV |
| 13/11/2023 | Killed | Yacoub Al-Borsh | CPJ - IFJ - SKeyes - SHFA | M | Home | Staff | Namaa Radio |
| 18/11/2023 | Killed | Abdelhalim Awad | CPJ - IFJ - PJS – Skeyes | M | Home | Staff | Al-Aqsa TV |
| 18/11/2023 | Killed | Amro Salah Abu Hayah | CPJ - IFJ - PJS - Al-Jazeera | M | Airstrike | Staff | Al Aqsa TV |
| 18/11/2023 | C* | Hassouneh Salim | CPJ - IFJ -PJS Al-Jazeera | M | Airstrike | Freelance |  |
| 18/11/2023 | C | Mossab Ashour | CPJ - IFJ - PJS - Al-Jazeera | M | Airstrike | Freelance |  |
| 18/11/2023 | C | Mostafa El Sawaf | CPJ - IFJ - PJS - SKeyes | M | Home | Staff | MSDR News |
| 18/11/2023 | C* | Sari Mansour | CPJ - IFJ - PJS - Al-Jazeera | M | Home | Staff | Quds News Network |
| 19/11/2023 | C* | Bilal Jadallah | CPJ - IFJ - PJS - Al Qahera | M | Targeted | Staff | Press House-Palestine |
| 20/11/2023 | C* | Ayat Khadoura | CPJ - IFJ - SKeyes - Arabi 21 | F | Home | Freelance |  |
| 21/11/2023 | C | Jamal Mohamed Haniyeh | CPJ - IFJ – PJS | M | Home | Staff | Amwaj |
| 22/11/2023 | Killed* | Assem Al-Barsh | CPJ - IFJ – PJS | M | Sniper | Staff | Al-Rai |
| 22/11/2023 | C | Mohamed Nabil Al-Zaq | CPJ - IFJ - PJS - Roya | M | Home | Staff | Al Quds |
| 23/11/2023 |  | Amal Zahed (Zohd) | IFJ, CFWIJ, Al-Mayadeen | F | Home |  | Al-Jazeera, Al Mayadeen |
| 23/11/2023 | C | Mohamed Mouin Ayyash | CPJ - Roya - PJS – Wafa | M | Home | Freelance |  |
| 24/11/2023 | C | Mostafa Bakeer | CPJ - IFJ - PJS - Al-Aqsa | M | Home | Staff | Al Aqsa TV |
| 1/12/2023 | C | Abdullah Darwish | CPJ - IFJ - PJS – Roya | M | Airstrike | Staff | Al Aqsa TV |
| 1/12/2023 | C | Adham Hassouna | CPJ - SHFA - SKeyes – PJS | M | Home | Freelance |  |
| 1/12/2023 | C* | Montaser Al-Sawaf | CPJ -IFJ - Anadolu - Middle East Monitor | M | Airstrike | Staff | Anadolu Agency |
| 1/12/2023 |  | Marwan Al Sawaf | CPJ - IFJ - PJS | M | Airstrike | Staff | Alef multimedia |
| 3/12/2023 | Killed | Hamada Al-Yaziji | CPJ - SKeyes | M | Airstrike | Staff | Al Quds - Kanaan |
| 3/12/2023 | Killed | Hassan Farajallah | CPJ - IFJ – PJS | M | Airstrike | Staff | Al Quds TV |
| 3/12/2023 | C | Shaima El-Gazzar | CPJ - SKeyes – Darb | F | Home | Staff | Al-Majedat network |
| 9/12/2023 | C | Duaa Jabbour | CPJ - PJS - Anadolu - Al-Araby Al-Jadeed | F | Home | Freelance |  |
| 9/12/2023 | C | Ola Atallah | CPJ - PJS - Anadolu Agency - Arabi 21 | F | Home | Freelance |  |
| 15/12/2023 | C* | Samer Abu Daqqa | CPJ - Al-Jazeera | M | Airstrike | Staff | Al-Jazeera Arabic |
| 17/12/2023 | C | Assem Kamal Moussa | CPJ - Al-Mayadeen - Al-Aqsa | M | Airstrike | Staff | Palestine Now |
| 17/12/2023 | C | Haneen Kashtan | CPJ - PJS - Al-Jazeera | F | Home | Staff | TV Al-Kofiya, Baladna Web TV |
| 18/12/2023 | C | Abdallah Alwan | CPJ - Midan - Al-Aqsa - Al-Hadath | M | Home | Staff | Holy Quran Radio, Midan, Mugtama, Al-Jazeera |
| 19/12/2023 | C | Adel Zorob | CPJ - PJS - WAFA - Middle East Eye | M | Home | Freelance |  |
| 22/12/2023 | Killed | Mohamed (Saidi) Khalifeh | CPJ - Anadolu - Al -Mayadeen TV - Al-Ghad TV | M | Home | Staff | Al Aqsa TV |
| 23/12/2023 | C | Mohamed Naser Abu Huwaidi | CPJ - PJS - SKeyes - Al-Araby Al-Jadeed | M | Airstrike | Staff | Al-Istiklal |
| 24/12/2023 | C | Ahmad Jamal Al Madhoun | CPJ - Quds News Network - Al-Araby Al-Jadeed - Anadolu Agency | M | Airstrike | Staff | Al-Rai |
| 24/12/2023 | C | Mohamad Al-Iff (Mohammad Abdul Khaleq Al Ghuf) | CPJ - SKeyes - Al-Araby Al-Jadeed - Quds News Network | M | Home | Staff | Al-Rai |
| 24/12/2023 | Killed | Mohamed Azzaytouniyah | CPJ - Al-Araby Al-Jadeed - Quds News Network | M | Home | Staff | Al-Rai |
| 28/12/2023 | C | Ahmed Khaireddine (Ahmad Khair Al Din) | CPJ - PJS - Al-Jazeera - Palestine Today | M | Home | Staff | Quds Al-Youm TV, Al Aqsa TV |
| 29/12/2023 | C | Jabr Abu Hadrous | CPJ - Al-Jazeera | M | Home | Staff | Quds Al-Youm |
| 5/1/2024 | C | Akram ElShafie | CPJ - IFJ | M | Hospital (injured October 30) | Staff | Safa |
| 7/1/2024 | C* | Hamza Al Dahdouh | CPJ - PJS - Al-Jazeera - New York Times; Channel 4 News - witnesses | M | Targeted | Staff | Al-Jazeera |
| 7/1/2024 | C* | Mustafa Thuraya | CPJ - PJS - Al-Jazeera - New York Times; Channel 4 News - witnesses | M | Targeted | Freelance |  |
| 8/1/2024 | C | Abdallah Iyad Breis | CPJ - SKeyes - WAFA | M | Home | Staff | Rawafed educational channel |
| 9/1/2024 | C | Heba Al-Abadla | CPJ - PJS - SKeyes - WAFA | F | Home | Staff | Al-Azhar radio station, owned by Al-Azhar University in Gaza |
| 10/1/2024 | C* | Ahmed Bdeir | CPJ - Al-Jazeera - The New Arab - SKeyes | M | Airstrike | Staff | Bawabat al-Hadaf |
| 10/1/2024 | C | Shareef Okasha | CPJ - SKeyes | M | Home | Freelance | Al Jazeera, Al Ghad, and Al Koofiya TV |
| 11/1/2024 | C | Mohamed Jamal Sobhi Al-Thalathini | CPJ - Al-Aqsa Voice Radio - Palestine Today - Al-Araby Al-Jadeed | M | Home | Staff | Al-Quds Al-Youm |
| 14/1/2024 | C* | Yazan al-Zuweidi (al-Zwaidi) | CPJ - PJS - SKeyes - The New Arab | M | Airstrike | Staff | Al-Ghad |
| 25/1/2024 | C | Iyad El-Ruwagh (Iyad Ahmed Al-Ruwahi) | CPJ - IFJ - PJS - Safa | M | Home | Staff | Al-Aqsa Voice Radio |
| 29/1/2024 | C | Mohammed Atallah (Mohamed Abdel El Fatah Atta Allah) | CPJ - SKeyes - WAFA | M | Home (Refugee camp) | Staff | Al-Risalah, Raseef 22 |
| 29/1/2024 | C | Tariq Al-Maidna | CPJ - SKeyes | M | Targeted | Freelance | Al Houthi TV |
| 6/2/2024 | C | Rizq Al-Gharabli | CPJ - Palestinian Information Center - SKeyes - WAFA | M | Home | Staff | Palestinian Information Center |
| 8/2/2024 | C | Nafez Abdel Jawad | CPJ - IFJ - CNN - Anadolu Agency | M | Home | Staff | Palestine TV (official Palestine Television station) |
| 11/2/2024 | C* | Yasser Mamdouh El-Fady | CPJ - SKeyes | M | Sniper | Staff | Kan’an news agency |
| 12/2/2024 | C | Alaa Al-Hams | CPJ - IFJ - PJS - Palestine Chronicle - Ahram | F | Home | Staff | Palestinian News Agency SND |
| 12/2/2024 | C | Angam Ahmad Edwan | CPJ - IFJ - Ahram - Anadolu - Palestine Chronicle | F | Home | Staff | February  (Libyan TV channel) |
| 14/2/2024 |  | Ayman Al-Rafati | CPJ - Al Jazeera -The Middle East Eye - Quds News Network. | M | Home | Staff | Al Mayadeen |
| 15/2/2024 | C | Zayd Abu Zayed | CPJ - Anadolu - WAFA | M | Airstrike | Staff | Quran Radio channel, owned by the Islamic University in Gaza |
| 23/2/2024 | C | Mohamed Yaghi | CPJ - IFJ - PJS | M | Airstrike | Staff | Al-Jazeera |
| 5/3/2024 | C | Muhammad Salama (Mohammed Khader Ahmad Salama) | CPJ - IFJ - PJS - Anadolu Agency | M | Home | staff | Al Aqsa TV |
| 14/3/2024 | C | Abdel Rahman Saima | CPJ - IFJ | M | Airstrike | Staff | Raqmi TV |
| 15/3/2024 | C | Mohamed el-Reefi (Al-Rifi) | CPJ - IFJ | M | Airstrike | Freelance |  |
| 18/3/2024 | C | Tarek El Sayed Abu Skheil | CPJ - IFJ | M | Airstrike | Staff | Al-Quds |
| 25/3/2024 | C | Saher Akram Rayyan | CPJ - IFJ - WAFA | M | Airstrike | Staff | WAFA news agency |
| 28/3/2024 | C | Mohammed Abu Skheil(Sakhil ) | CPJ - IFJ - Shams News Agency | M | Home (Refugee in hospital) | Staff | Shams News Agency |
| 25/4/2024 | C | Mohammed Bassam Al Jamal | CPJ - PJS - SKeyes - Al-Ghad TV channel - Al-Araby Al-Jadeed | M | Home | Staff | Palestine Now |
| 26/4/2024 | C | Ayman Mohamad Al-Gharbawi | CPJ - SKeyes - Shfa - RT - WAFA | M | Strike § | Freelance |  |
| 26/4/2024 | C | Ibrahim Mohamad Al-Gharbawi | CPJ - SKeyes - Shfa - RT - WAFA | M | Strike § | Freelance |  |
| 29/4/2024 | C | Salem Abu Toyour | CPJ - Al Jazeera - SKeyes - Wattan - WAFA | M | Home | staff | Al-Quds Al-Youm TV channel |
| 31/3/2024 | C | Mustafa Bahr | CPJ - SKeyes | M | Airstrike | Staff | Palestine Breaking News website |

**Legend:**

C: Confirmed.

C*“According to the information collected by RSF, may have been deliberately targeted as journalists” and for which RSF has filed war crimes complaints with the International Criminal Court (ICC) (<https://rsf.org/en/rsf-files-second-complaint-icc-war-crimes-against-journalists-gaza-7-october>).

§ On assignment.

M: Male.

F: Female.

*Confirmed*: “CPJ considers a case “confirmed” as work-related only when it appears certain that a journalist was murdered in direct reprisal for his or her work; in combat or crossfire; or while carrying out a dangerous assignment. Cases involving unclear motives, but with a potential link to journalism, are classified as “unconfirmed” and CPJ continues to investigate. The “unconfirmed” category does not include journalists who are killed in accidents or other incidents where the journalist was not on assignment and there is no evidence to suggest the journalist was the target.”

# S3_2. Table S2. Cross-Tabulation between journalist lists

| List Capture | N (%) |
| --- | --- |
| GMO only | 34 (22%) |
| CPJ only | 11 (7%) |
| IFJ only | 2 (1%) |
| GMO captured only in CPJ | 15 (10%) |
| GMO captured only in IFJ | 9 (6%) |
| IFJ captured only in CPJ | 4 (2%) |
| GMO captured in both other lists | 82 (52%) |
|  |  |
| Total | 157 (100%) |

GMO=Government Media Office; CPJ=Committee to Protect Journalists; IFJ=International Federation of Journalists;

**GMO list n=140; IFJ list n=106; CPJ list n=103; Total unique deaths =157**

# S3_3. Table S3. Estimates from fitting a log-linear model on the counts

| Factor | Estimate | Std error | p-value |
| --- | --- | --- | --- |
|  |  |  |  |
| GMO | -3.73 | 0.67 | 0.0001 |
| CPJ | -1.33 | 0.37 | 0.0004 |
| IFJ | -0.20 | 0.45 | 0.66 |
| CPJ*GMO | 2.98 | 0.62 | 0.0001 |
| CPJ*IFJ | 2.95 | 0.46 | 0.0001 |

GMO=Government Media Office; CPJ=Committee to Protect Journalists; IFJ=International Federation of Journalists;

**Predicted additional deaths not present in any of the list according to the model =44, total = 201.**
